# Three‐Phase Electrolysis by Gold Nanoparticle on Hydrophobic Interface for Enhanced Electrochemical Nitrogen Reduction Reaction

**DOI:** 10.1002/advs.202002630

**Published:** 2020-10-12

**Authors:** Junchang Zhang, Bo Zhao, Wenkai Liang, Genshu Zhou, Zhiqiang Liang, Yawen Wang, Jiangying Qu, Yinghui Sun, Lin Jiang

**Affiliations:** ^1^ School of Environment and Civil Engineering Dongguan University of Technology Dongguan Guangdong 523808 China; ^2^ School of Chemistry and Chemical Engineering Nantong University Nantong Jiangsu 226019 China; ^3^ Institute of Functional Nano and Soft Materials (FUNSOM) Jiangsu Key Laboratory for Carbon‐Based Functional Materials and Devices Soochow University Suzhou Jiangsu 215123 China; ^4^ State Key Laboratory for Mechanical Behavior of Materials Xi'an Jiaotong University Xi'an Shanxi 710049 China; ^5^ College of Energy Soochow Institute for Energy and Materials Innovations and Key Laboratory of Advanced Carbon Materials and Wearable Energy Technologies of Jiangsu Province Soochow University Suzhou Jiangsu 215006 China

**Keywords:** electrochemical nitrogen reduction, gold nanoparticles, hydrophobic interfaces, three‐phase contact points

## Abstract

Electrochemical nitrogen reduction reaction (NRR) provides a facile and sustainable strategy to produce ammonia (NH_3_) at ambient conditions. However, the low NH_3_ yield and Faradaic efficiency (FE) are still the main challenges due to the competitive hydrogen evolution reaction (HER). Herein, a three‐phase electrocatalyst through in situ fabrication of Au nanoparticles (NPs) located on hydrophobic carbon fiber paper (Au/o‐CFP) is designed. The hydrophobic CFP surface facilitates efficient three‐phase contact points (TPCPs) for N_2_ (gas), electrolyte (liquid), and Au NPs (solid). Thus, concentrated N_2_ molecules can contact the electrocatalyst surface directly, inhibiting the HER since the lowered proton concentration and overall enhancing NRR. The three‐phase Au/o‐CFP electrocatalyst presents an excellent NRR performance with high NH_3_ yield rate of 40.6 µg h^−1^ mg^−1^ at −0.30 V and great FE of 31.3% at −0.10 V versus RHE (0.1 m Na_2_SO_4_). The N_2_‐bubble contact angle result and cyclic voltammetry analysis confirm that the hydrophobic interface has a relatively strong interaction with N_2_ bubble for enhanced NRR and weak electrocatalytic activity for HER. Significantly, the three‐phase Au/o‐CFP exhibits excellent stability with a negligible fluctuation of NH_3_ yield and FE in seven‐cycle test. This work provides a new strategy for improving NRR and simultaneously inhibiting HER.

## Graphical abstract

A three‐phase electrocatalyst with abundant three‐phase contact points (TPCPs) is achieved by direct in situ fabrication of Au NPs on hydrophobic carbon fiber electrode, demonstrating high ammonia yield rate (40.6 µg h^−1^ mg^−1^) and high Faradaic efficiency (31.3%).

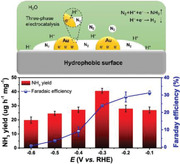

## 1 Introduction

Ammonia (NH_3_) as one of the most important chemical products plays a key role in many areas, including agricultural fertilizer, textile industries, and medicaments.^[^ ^1^, ^2^, ^3^, ^4^ ^]^ Moreover, NH_3_ is also drawing considerable attention due to its advantages of high‐efficiency carbon‐free energy carrier with high hydrogen density as well as convenient storage and transportation at room temperature.^[^ ^5^, ^6^, ^7^ ^]^ Presently, the production of NH_3_ is mainly relied on the industrial Haber‐Bosch (H‐B) process by coactivation of nitrogen (N_2_) and hydrogen (H_2_) over Fe‐based catalysts under high temperature and high pressure (300–550 °C and 200–350 atm).^[^ ^8^, ^9^, ^10^ ^]^ Unfortunately, over 1% of the global energy supply annually is consumed during the H–B process because of the high N≡N bond dissociation energy.^[^ ^11^, ^12^, ^13^, ^14^ ^]^ Meanwhile, large amounts of green‐house gases are produced. Therefore, it is extremely urgent to explore a facile and sustainable approach for NH_3_ production.

Encouragingly, the electrochemical ammonia synthesis provides an alternative method because of the merits of low cost and friendly to environment.^[^ ^15^, ^16^, ^17^ ^]^ It has been proved theoretically and experimentally that noble metal catalysts, such as Au,^[^ ^18^ ^]^ Pt,^[^ ^19^, ^20^ ^]^ and Ru,^[^ ^21^, ^22^ ^]^ represent better NRR activities superior to non‐metal catalysts, owing to the stronger N‐H bonding and higher electrical conductivity. Specially, Au is arguably one of the most promising catalysts for the electrochemical N_2_ reduction reaction. Recently, researchers have improved the NH_3_ yield and Faraday efficiency by modifying Au morphology,^[^ ^23^, ^24^, ^25^ ^]^ crystalline structure^[^ ^26^ ^]^ and crystal facet.^[^ ^18^, ^27^ ^]^ However, the low yield and Faradaic efficiency are still far from ideal performance of NRR due to competitive hydrogen evolution reaction (HER).^[^ ^13^ ^]^ Therefore, it is an urgent topic to develop an electrocatalytic system that can simultaneously promote the N—H reaction for NRR and suppress the reduction of proton for HER.

One fact cannot be ignored is extremely low solubility of N_2_ in electrolyte, thus limiting the supply of N_2_ molecules to the catalyst surface by their low concentration and slow diffusivity.^[^ ^28^ ^]^ Meanwhile, the protons (H^+^) are readily available in aqueous solutions by water ionization, resulting in overwhelmingly competitive HER. Therefore, a promising strategy is the increase of N_2_ concentration and the reduction of H^+^ concentration on the catalyst surface to improve NRR and suppress HER. Recently, the hydrophobic interface is highly appealing in gas‐involved electrochemical reactions because it can provide abundant three‐phase contact points (TPCPs) for gas, electrolyte (liquid) and catalyst (solid). Additionally, the hydrophobic interface affords a fast gas diffusion path, resulting in sufficient supply of gas to the catalyst surface.^[^ ^29^, ^30^, ^31^, ^32^ ^]^ Thus, electrochemical gas evolution reactions prefer to operate on TPCPs located at the hydrophobic surface rather than wetted by the electrolyte.^[^ ^29^ ^]^ Moreover, the H^+^ concentration at the hydrophobic interface becomes lower than hydrophilic interface due to the insufficient contact between water and the hydrophobic interface.^[^ ^30^, ^33^ ^]^ Therefore, designing a three‐phase electrocatalyst at hydrophobic interface should be an effective method to reinforce NRR and suppress HER. So far, only Ling et al. and Du et al. just focused on the surface modification of electrocatalysts by the introduce of a hydrophobic zeolitic imidazolate frameworks to cover Au or Au–Ag forming a three‐phase interface for suppressed HER and enhanced electrochemical NRR.^[^ ^34^, ^35^ ^]^ However, the continuous hydrophobic shell prevented the available and sufficient contact of N_2_, electrolyte and catalyst. Moreover, the complicated synthesis and fabrication process largely hinder its practical applications and commercial viability. To the best of our knowledge, there are no reports on the forming of a three‐phase electrocatalyst by the hydrophobic interface of the support electrode for NRR.

Herein, we report an artful strategy to design a three‐phase electrocatalyst with abundant TPCPs by direct in situ fabrication of Au nanoparticles (NPs) on hydrophobic carbon fiber electrode. A superior ammonia yield of 40.6 µg h^−1^ mg^−1^ at −0.3 V versus reversible hydrogen electrode (RHE) and Faradaic efficiency of 31.3% at −0.1 V versus RHE were achieved, which are much higher than those of Au/i‐CFP. Notably, the Au/o‐CFP also showed outstanding stability with a negligible fluctuation of electrocatalytic activity after seven recycles. N_2_‐bubble contact angle result analysis showed that Au/o‐CFP possesses an aerophilic property with a relatively strong interaction toward N_2_ bubble, leading to significant increased NRR. Additionally, the low H^+^ concentration at the hydrophobic interface resulted in suppressed HER, which is also confirmed by cyclic voltammetry (CV) analysis.

## 2 Results and Discussion

**Figure 1:**
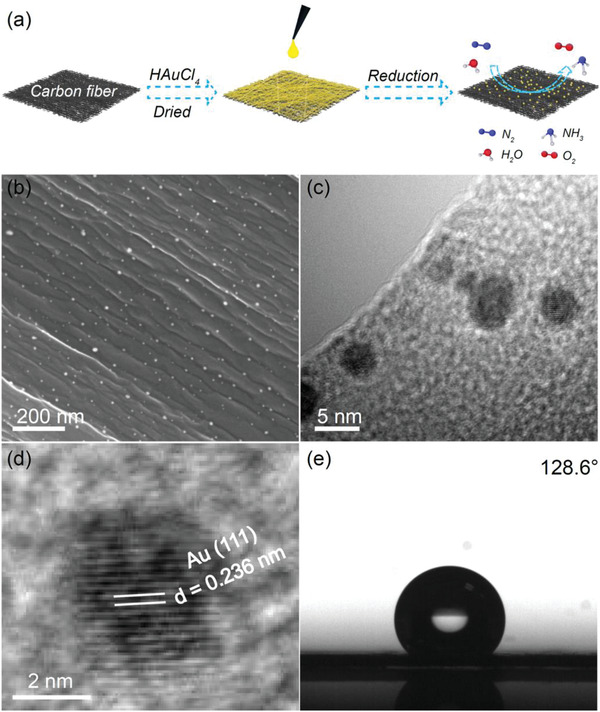
a) Schematic diagram for illustrating the fabrication of Au/o‐CFP and electrocatalytic nitrogen reduction to ammonia; b) high resolution SEM image of Au NPs on carbon paper; c,d) HRTEM images of Au NPs; e) water contact angle measurement of Au/o‐CFP.

The three‐phase electrocatalysts presented here is a hydrophobic carbon fiber paper with loading of Au NPs (Au/o‐CFP), which was simply fabricated by in situ reduction of HAuCl_4_ on carbon fiber paper. As shown in **Figure** **1**a, the HAuCl_4_ solution firstly overspread the carbon fiber paper. After drying, the carbon fiber paper was immersed into NaBH_4_ solution for 1 min. Then, well‐dispersed Au NPs formed on the carbon fiber. Scanning electron microscopy (SEM) reveals that Au NPs display spherical morphology and are uniformly distributed on the surface of carbon fiber (Figure 1b). The average size of Au NPs is about 6.8 nm, which is counted based on transmission electron microscopy (TEM) images (Figure S1, Supporting Information). The size of the obtained Au NPs depends on the amount of HAuCl_4_, where the higher volume of HAuCl_4_, the larger size of Au (Figure S1, Supporting Information). A lattice spacing of 0.236 nm is observed from the high‐resolution TEM (HRTEM) image, indicating the (111) interplanar distance of the face centered cubic (fcc) Au crystal (Figure 1c,d). X‐ray photoelectron spectroscopy (XPS) was performed to study the chemical state of Au NPs. The result of Au 4f spectrum shows two peaks at 84 and 87.7 eV (Figure S2, Supporting Information), which can be ascribed to 4f_7/2_ and 4f_5/2_ electrons, respectively. Contact‐angle measurement was carried out to study the surface property relating to water wetting. The average contact angle of Au/o‐CFP surface is 128.6° (Figure 1e), indicating the electrode possesses a significant hydrophobicity.

**Figure 2:**
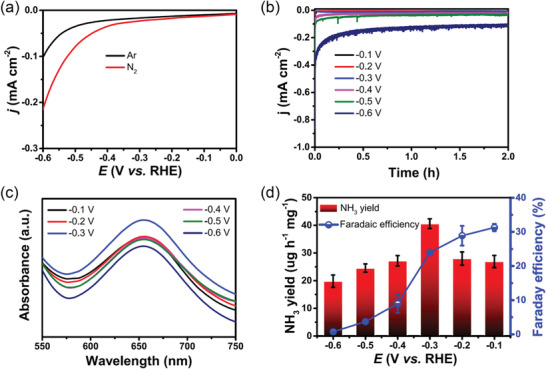
LSV curves in Ar‐ or N_2_‐ saturated 0.1 M Na_2_SO_4_ solution under ambient condition; b) the chronoamperometry tests with Au/o‐CFP electrode at various potentials from −0.60 to −0.10 V versus RHE; c) corresponding UV–vis spectra of electrolytes colored with indophenol indicator; d) ammonia yield rates and Faradaic efficiencies at various potentials.

The NRR performance was performed in an H‐type cell with three‐electrode system. The Au/o‐CFP was directly used as a working electrode. All potentials in the test were converted into the potentials versus reversible hydrogen electrode (RHE). The sweep voltammetry (LSV) curves were conducted in Ar‐ and N_2_‐saturated 0.1 m Na_2_SO_4_ aqueous solution (pH = 7). As shown in **Figure** **2**a, the curve between −0.1 and −0.6 V in N_2_ shows an obvious difference with that in Ar, which suggests a significant response of Au/o‐CFP to N_2_. Thus, it is proposed that the maximal NH_3_ yield can be obtained in the voltage range. The chronoamperometry tests were further used to determine the NH_3_ yield and Faraday efficiency of Au/o‐CFP. The time‐depended current density curves in N_2_‐satutated electrolyte are showed in Figure 2b and all the curves of Au/o‐CFP holds stable at different potentials for 2 h, suggesting a good durability of Au/o‐CFP. Concentrations of NH_3_ production and hydrazine (N_2_H_4_) were analyzed by UV–Vis spectroscopy (Section 4), and the corresponding standard curves were displayed in Figures S3 and S4, Supporting Information. The UV absorption spectra of the electrolyte after 2 h reaction are displayed in Figure 2c. Notably, the highest absorbance value (655 nm) at −0.30 V was achieved, indicating the maximal NH_3_ yield at this potential on the Au/o‐CFP. According to the standard curve, the average NH_3_ yield and Faraday efficiency are further calculated as shown in Figure 2d. Significantly, the highest NH_3_ yield rate of 40.6 µg h^−1^ mg^−1^ is obtained at −0.30 V versus RHE and the greatest Faradaic efficiency of 31.3% is achieved at −0.10 V versus RHE. More importantly, this efficiency is superior to most of recently reported NRR electrocatalysts, such as B doped Ag NPs (26.48 µg h^−1^ mg^−1^_cat._, 8.86%),^[^ ^36^ ^]^ porous Au film (29.43 µg h^−1^ mg^−1^_cat._, 13.36%)^[^ ^37^ ^]^ and sub‐cluster Au/TiO_2_ (21.4 µg h^−1^ mg^−1^_cat._, 8.11%).^[^ ^38^ ^]^ The detailed information is displayed in Table S1, Supporting Information. In order to further explore the selectivity of product by NRR, the possible by‐product of N_2_H_4_ was also analyzed. Remarkably, no N_2_H_4_ is detected in 0.1 m Na_2_SO_4_ solution after 2 h electrolysis at different potentials (Figure S5, Supporting Information), suggesting 100% selectivity for reducing N_2_ to NH_3_ on the Au/o‐CFP.

**Figure 3:**
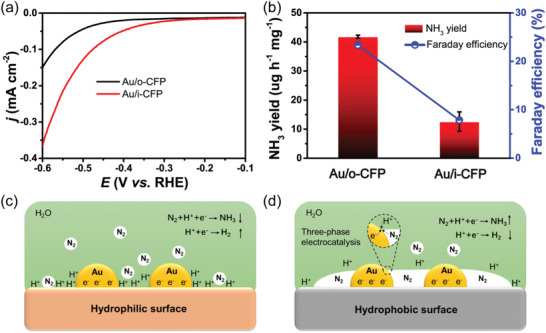
a) LSV curves of Au/o‐CFP and Au/i‐CFP in Ar‐saturation electrolyte; b) ammonia yield rates and Faradaic efficiencies at −0.30 V for Au/o‐CFP and Au/i‐CFP. The schematic illumination three‐phase contact for N_2_ (gas), electrlyte (liquid), and catalyst (solid) at c) hydrophilic interface and d) hydrophobic interface.

In order to verify the effect of surface property wetting to water of carbon fiber electrode on NRR performance, Au/o‐CFP was treated by O_2_ plasma to achieve hydrophilic surface of CFP (Au/i‐CFP). After the treatment, the oxygen functional groups on the interface increases, confirmed by XPS spectra (Figure S6, Supporting Information). The contact angle of Au/o‐CFP electrode changed from 128.6° to 0°, indicating the electrode possessing a remarkable hydrophilicity. The morphology of Au electrocatalysts was kept well (Figure S7, Supporting Information) after O_2_ plasma treatment. **Figure** **3**a shows the LSV curves of Au/o‐CFP and Au/i‐CFP in Ar‐saturated electrolyte. It is obvious that the current density of Au/o‐CFP is much lower than that of Au/i‐CFP at the same driven voltage, suggesting fewer protons involved in electrolysis, resulting in reduced HER.^[^ ^35^, ^39^ ^]^ To verify the effect of hydrophobic interface on suppressed HER, we calculate the electrochemically active surface areas by extracting the double layer capacitance (*C*_dl_),^[^ ^40^, ^41^ ^]^ which is investigated by the cyclic voltammetry (CV) measurements in the range of −0.2 and −0.4 V under Ar condition (Figure S8, Supporting Information). The *C*_dl_ value of Au/o‐CFP is calculated to be 0.085 mF cm^−2^, which is much lower than that of Au/i‐CFP (1.15 mF cm^–2^), demonstrating the lower electrocatalytic activity of Au/o‐CFP for HER. This result can be ascribed to the insufficient contact area between water and hydrophobic surface.^[^ ^32^ ^]^ Furthermore, the average NH_3_ yield rate and Faradaic efficiency conducted at −0.30 V are detected. As shown in Figure 3b, the Au/i‐CFP exhibits a much lower NRR performance (12.6 µg h^−1^ mg^−1^, 7.86%), which is below the half of that for Au/o‐CFP NRR performance. These results demonstrate the hydrophobic interface play a key role for enhanced NRR activity and suppressed HER activity for Au/o‐CFP. Based on above results, a proposed mechanism for enhanced NRR performance and suppressed HER activity of Au/o‐CFP is shown in Figure 3c,d. Because of the extremely low solubility and slow diffusion rate of N_2_ and easily available H^+^, the supply of high concentrations of N_2_ molecules to catalyst surface and limiting the concentration of H^+^ becomes more important in aqueous solution. The hydrophilic interface provides sufficient contact between catalyst and water, resulting in more absorbed H^+^ (Figure 3c). Moreover, the Au/i‐CFP shows a high gas‐bubble contact angle (CA_g_) of 141.5° (Figure S9a, Supporting Information), implying a weak interaction with N_2_ bubble. Consequently, the concentration of N_2_ molecule is low and the concentration of H^+^ is high near the surface of catalyst, resulting in enhanced HER and suppressed NRR. By contrast, the hydrophobic interface provides sufficient N_2_ with a fast diffusion pathway and H^+^ with relative low concentration due to the hydrophobic property.^[^ ^29^, ^30^, ^31^, ^32^ ^]^ Moreover, the surface of Au/o‐CFP with a gas‐bubble contact angle (CA_g_) of 112° provided an aerophilic property with a relatively strong interaction toward N_2_ bubble (Figure S9b, Supporting Information), resulting in sufficient supply of N_2_ to catalyst. Therefore, abundant three‐phase contacts for N_2_, water and Au form (Figure 3d), resulting in enhanced NRR performance and suppressed HER performance.

**Figure 4:**
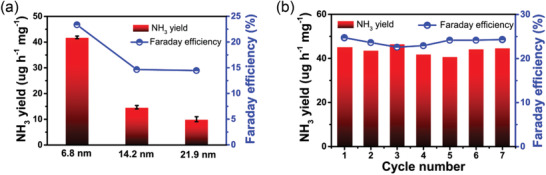
a) Comparison of catalytic performances with the size of Au NPs increasing; b) Cycling tests of Au/o‐CFP catalyst at −0.30 V versus RHE.

To confirm the origin of NH_3_, three controlled experiments were conducted: i) the working electrode in electrolyte with continual Ar flow at −0.30 V for 2 h electrolysis; ii) the working electrode in electrolyte with continual N_2_ flow at open circuit potential for 2 h electrolysis; iii) bare carbon paper in electrolyte with continual N_2_ flow at −0.30 V for 2 h electrolysis. Significantly, the corresponding UV–vis absorption spectra (Figure S10, Supporting Information) indicate that negligible product of NH_3_ is generated for the three conditions. The ^15^N isotopic labeling experiment is further conducted to verify the original N source of the NH_3_. As shown in Figure S11, Supporting Information, the spectra indicate a triplet coupling for ^14^NH_4_^+^ and a doublet coupling for ^15^NH_4_^+^ when using ^14^N_2_ and ^15^N_2_ as gas seed, which is consistent with that for (^14^NH_4_)_2_SO_4_ and (^15^NH_4_)_2_SO_4_. This result demonstrates the produced NH_3_ resulted from the electrocatalytic NRR on Au/o‐CFP. Furthermore, the particle size effect of Au NPs on NRR performance on Au NPs was also analyzed at −0.30 V versus RHE. As shown in **Figure** **4**a, the NH_3_ yield rate and Faradaic efficiency decreases with the size increases, suggesting the size of Au may be another important factor for its high electrocatalytic activity for NRR.^[^ ^38^, ^42^, ^43^ ^]^ Additionally, the durability of electrocatalyst is an important factor for the practical application. Figure S12, Supporting Information illustrates the long‐term stability measured by chronoamperometry at −0.30 V. There is only a slight decrease in current density after 24 h electrocatalysis. After the test, the Au/o‐CFP exhibits a negligible decay of NH_3_ yield and Faradaic efficiency. Furthermore, the XPS spectra for Au in the 4f region is almost unchanged after 24 h electrocatalysis, demonstrating its remarkable stability (Figure S2, Supporting Information). More significantly, Au/o‐CFP shows a minor change in NH_3_ yield rate and Faradaic efficiency during seven times recycling tests (Figure 4b), indicating an excellent durability of Au/o‐CFP for NRR.

In this work, no N_2_H_4_ is detected during the electrocatalytic NRR in 0.1 m Na_2_SO_4_. Therefore, a proposed NRR mechanism of Au/o‐CFP is described in Figure S13, Supporting Information. Owing to unsaturated coordination, N_2_ molecule is weakly chemisorbed on Au NPs surface to form Au—N_2_ bond. Continuously, the nitrogen atom, which is far away from Au surface, is more likely to attract one proton, leading to the formation of N—H bond and the breaking of N≡N bond. Then after binding with another two protons in sequence, one NH_3_ molecule could be released. Finally, the remaining Au≡N bond is further hydrogenated like the above process to generate the second NH_3_ molecule. On basis of these results, the NRR mechanism in our case could be accepted and confirmed as a distal associative pathway.^[^ ^37^, ^44^ ^]^

## 3 Conclusion

In summary, we have demonstrated that Au/o‐CFP can be adopted as highly efficient NRR electrocatalysts, which taking the advantage of hydrophobic CFP support, promoting the efficient three‐phase contact of N_2_ gas, H_2_O (liquid), and Au (solid). Thus, a high concentration of N_2_ molecules and low concentration of H^+^ can achieve on the catalyst surface due to the fast diffusion gas pathway, which enhances the NRR and simultaneously inhibits the HER. As expected, the three‐phase Au/o‐CFP electrocatalyst shows a high NH_3_ yield rate of 40.6 µg h^−1^ mg^−1^ and great Faradaic efficiency of 31.3%, much higher than those of Au/i‐CFP. It also exhibits an excellent stability after seven consecutive cycles. N_2_‐bubble contact angle analyses also demonstrate that Au/o‐CFP possesses a relatively strong interaction with N_2_ bubble, resulting in significantly enhanced NRR. Furthermore, the CV measurement result confirms that Au/o‐CFP has a smaller double layer capacitance in Ar‐saturated electrolyte, indicating a weak catalytic activity for HER. This work demonstrates a unique class of three‐phase electrocatalysts with enhanced performance for NRR and suppressed performance for HER.

## 4 Experimental Section

##### Materials and Chemicals

Chloroauric acid (HAuCl_4_, 99.9%) and sodium borohydride (NaBH_4_) were bought from Sigma‐Aldrich. Ammonium chloride (NH_4_Cl), salicylic acid, sodium citrate dehydrate (Na_3_Ct), sodium hypochlorite solution (NaClO), anhydrous sodium sulfate (Na_2_SO_4_), and Nafion (5 wt%) solution were purchased from Alfa‐Aesar. Hydrazine monohydrate (≥98%, N_2_H_4_∙H_2_O) and *p*‐(dimethylamino) benzaldehyde were obtained from Aladdin. Sodium nitroferricyanide dehydrate were bought from Macklin. Carbon paper was provided by Shenzhen Teensky Technology Co., Ltd. N_2_ and Ar gases with high purity (99.999%) were used for the electrochemical measurements. The deionized (DI) water (18.2 MΩ) was purified by a Millipore system.

##### In Situ Fabrication of Au NPs

In a typical process, HAuCl_4_ aqueous solution (15 µL, 5mm) was dropped on the carbon paper (1 × 1 cm^2^), following drying at an infrared lamp. Then, the carbon paper side with HAuCl_4_ was immersed into NaBH_4_ solution (20 mm) for 1 min under stirring (200 rpm). Finally, the carbon paper was washed several times by DI water and ethanol, and drying at infrared lamp for use. The Au NPs with larger sizes were synthesized by increasing the volume of HAuCl_4_ (45 and 75 µL) at the same concentration. The loading mass of Au NPs was displayed in Table S2, Supporting Information.

##### Characterizations

Scanning electron microscopy (SEM) with a FEI Quanta 200 F SEM spectrometer was used to characterize the morphology of catalysts. Transmission electron microscopy (TEM) images were investigated on a Tecnai GZF20 transmission electron microscope at a working voltage of 200 kV. The loading mass of Au NPs on electrode was determined by inductively coupled plasma–mass spectrometer (ICP‐MS) on a Bruker Aurora M90 ICP mass spectrometer. X‐ray photoelectron spectroscopy (XPS) measurement was studied on Thermo Fischer ESCALAB 250Xi (Al K*α*). The contact angle was performed on a Theta Lite tensiometer equipped with a Firewire digital camera. Furthermore, the nitrogen (N_2_)‐bubble CA with the volume of 2 µL was conducted by the captive bubble method (DataPhysics OCA).^[^ ^45^ ^]^

##### Electrochemical NRR Measurements

An electrochemical workstation (CHI 660) was used to conduct the electrochemical measurements. In which, the Au/o‐CFP was used as working electrode, Ag/AgCl electrode as reference electrode and Pt sheet as counter electrode, respectively. At the beginning, N_2_ was continually injected into the electrolyte (0.1 m Na_2_SO_4_) for half an hour. The sweep rate of all the LSV curves was set as 5 mV s^−1^. During the NRR tests, the chronoamperomertic measurements were conducted in N_2_‐saturated 0.1 m Na_2_SO_4_ solution at various potentials. And N_2_ gas was continuously injected to the electrolyte during all the process. CV curves were conducted between −0.2 and −0.4 V at the scan rates of 10, 20, 40, 60, 80, and 100 mV s^–1^. All potentials were transformed to a reversible hydrogen electrode (RHE) by the under equation: *E*_RHE_ = *E*_Ag/AgCl_ + 0.059 × pH + 0.21 V.

##### Production Detection

The indophenol blue method^[^ ^46^ ^]^ was used for determining the concentration of generated ammonia. First, 2 mL electrolyte was mixed with three solutions (A, B, and C) for 2 h at ambient condition. In which, A is 2 mL NaOH (1 m) solution containing 5 wt% salicylic acid and 5 wt% sodium citrate, B is 1 mL NaClO (0.05 m) and C is 0.2 mL C_5_FeN_6_Na_2_O (1 wt%). Then, an UV–vis spectrophotometer was used to detect the absorption spectra of mixed electrolyte. The absorbance of indophenol blue was detected at 655 nm. To obtain the ammonia yield, the concentration‐absorbance curve was getting by using NH_4_Cl with a range concentration (0–1 µg mL^−1^) as standards. The equation: *r*_NH3_ = *C*_NH3_ *× V/(t* × *m)* was used to calculate the ammonia yield. In which *c*_NH3_ (µg mL^–1^), *V* (mL), *t* (h), and *m* (mg) are the mass concentration of NH_3_, the volume of the Na_2_SO_4_ electrolyte, the electrolysis time and the mass of catalysts, respectively.

The method of Watt and Chrisp was used to determine the concentration of hydrazine in 0.1 m Na_2_SO_4_ electrolyte.^[^ ^47^ ^]^ In brief, the color reagent was a mixture of 5.99 g *p*‐(dimethylamino) benzaldehyde, 300 mL ethanol and 30 mL concentrated HCl. Following that 5 mL electrolyte was added into 5 mL color reagent and stir for 10 min at ambient condition. The absorbance of the mixture solution was determined at 455 nm. The yields of hydrazine were calculated from a concentration‐absorbance standard curve, which is obtained by detecting hydrazine monohydrate with a concentration range (0–1 µg mL^−1^).

The ^14^N and ^15^N isotopic experiments were conducted using ^14^N_2_ and ^15^N_2_ as feeding gases for nitrogen reduction reaction. Before test, ^14^N_2_ and ^15^N_2_ gases were purged through 1 mm H_2_SO_4_ solution and water to eliminate the potential contaminants. After 6‐h electrochemical NRR in 0.1 m Na_2_SO_4_ solution at −0.3 V versus RHE, the total electrolyte solution for four reactions was together concentrated to 2.0 mL. Then, this solution was mixed with H_2_SO_4_ and DMSO‐*d*_6_ solutions and used for ^1^H NMR measurement (Agilent DD2‐600).

##### Faradaic Efficiency Determination

The Faradaic efficiency was calculated by the following equation: FE = 3F *× n*_NH3_ */Q*, in which F (96 500 C mol^−1^), *n*_NH3_ and Q represents the Faradaic constant, the mole of generated NH_3_ and the total electric quantity during the NRR test, respectively.

## Conflict of Interest

The authors declare no conflict of interest.

## Supporting information

Supporting Information
