# Supplementary material for: Three‐Phase Electrolysis by Gold Nanoparticle on Hydrophobic Interface for Enhanced Electrochemical Nitrogen Reduction Reaction
Source: Adv Sci (Weinh). 2020 Oct 12;7(22):2002630. doi: 10.1002/advs.202002630 (PMC7675187; doi:10.1002/advs.202002630)
Supplement: Supplementary file 1 — Supporting Information [file ADVS-7-2002630-s001.pdf]

WILEY-VCH

© 2020 Wiley-VCH GmbH

ADVANCED SCIENCE
Open Access

Supporting Information

for *Adv. Sci.*, DOI: 10.1002/advs.202002630

Three-Phase Electrolysis by Gold Nanoparticle on Hydrophobic Interface for Enhanced Electrochemical Nitrogen Reduction Reaction

Junchang Zhang, Bo Zhao, Wenkai Liang, Genshu Zhou, Zhiqiang Liang, Yawen Wang, Jiangying Qu, Yinghui Sun,* Lin Jiang**

© 2020 Wiley-VCH GmbH

Supporting Information

Three-phase electrolysis by gold nanoparticle on hydrophobic interface for enhanced electrochemical nitrogen reduction reaction

Junchang Zhang, Bo Zhao, Wenkai Liang, Genshu Zhou, Zhiqiang Liang, Yawen Wang, Jiangying Qu, Yinghui Sun,* Lin Jiang**

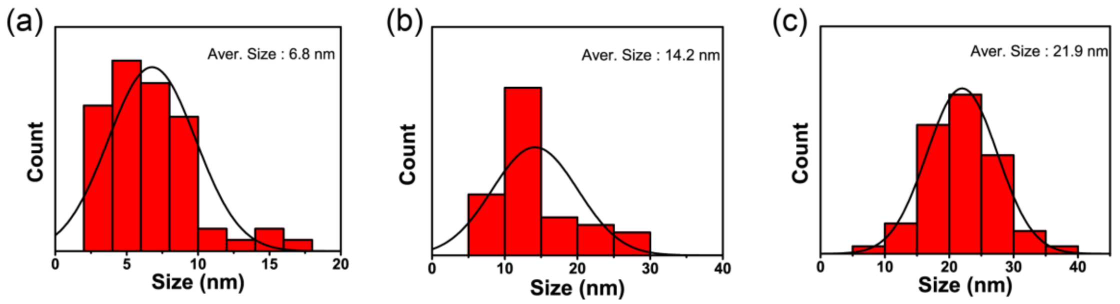

Figure S1. Size distribution diagram of Au NPs at different synthesis conditions of (a) 5 mM 15 μL, (b) 5 mM 45 μL and (c) 5 mM 75 μL.

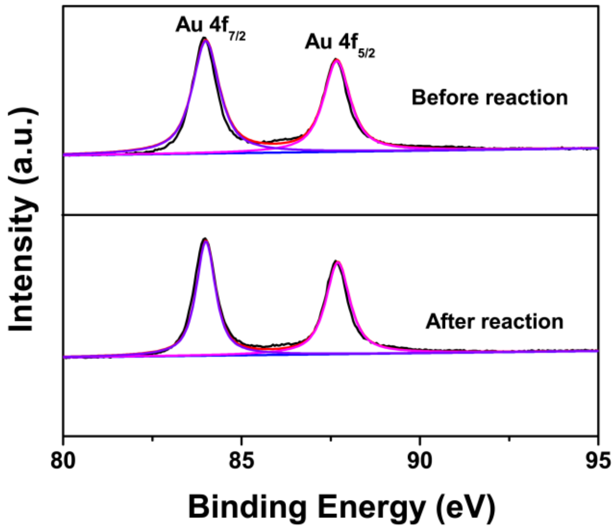

Figure S2. XPS spectrum of Au NPs in the Au 4f region before reaction and after 24-h electrolysis.

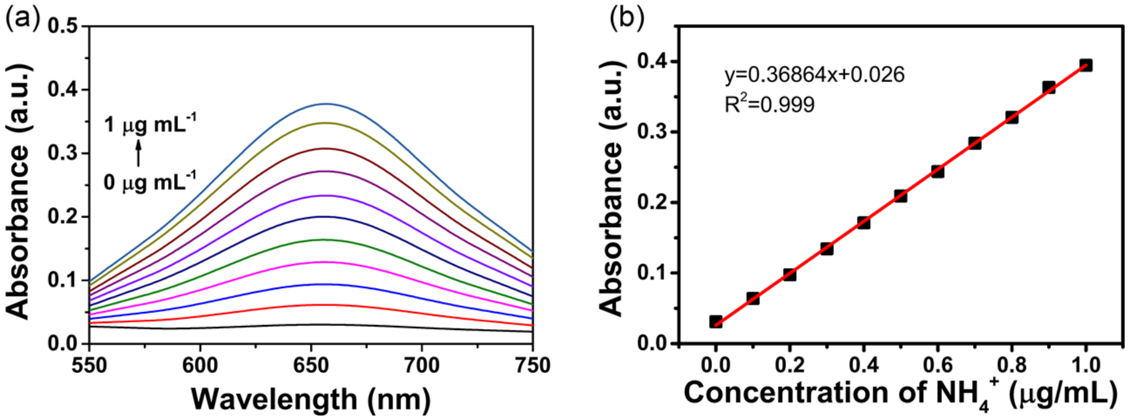

FS3. The UV-Vis absorption spectra and corresponding calibration curves for the colorimetric NH_3 assay using the indophenol blue method in 0.1 M Na_2SO_4.

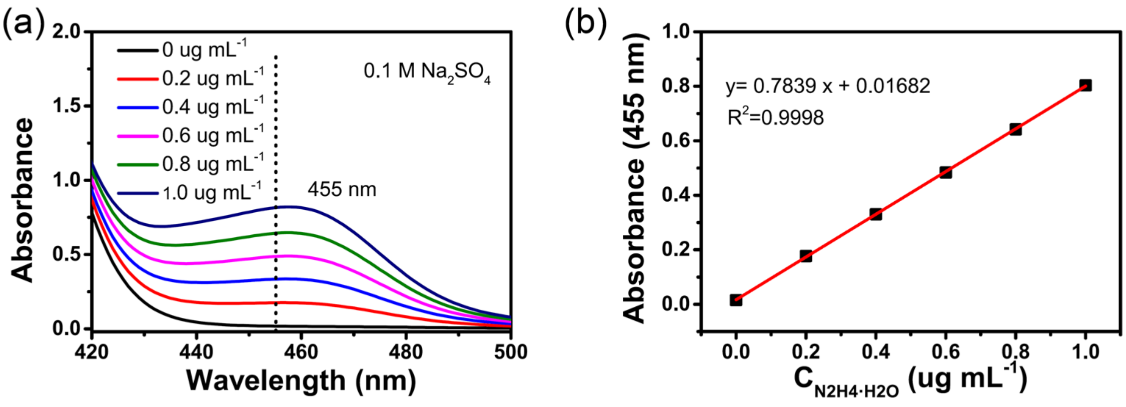

Figure S4. (a) The UV-Vis absorption spectra and (b) corresponding calibration curve for the colorimetric N_2H_4 assay in 0.1 M Na_2SO_4.

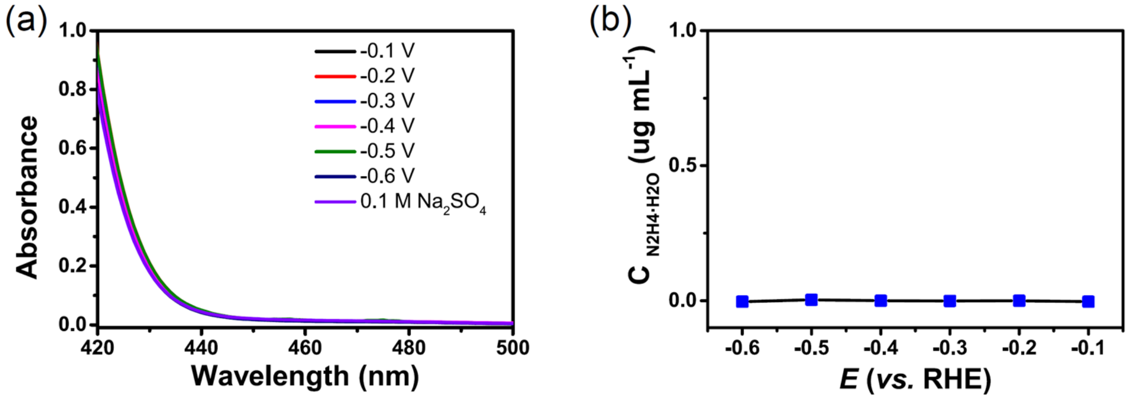

Figure S5. (a) The UV-Vis absorption spectra and (b) corresponding yield rate of $N_2H_4{\cdot}H_2O$ formation at selected potentials.

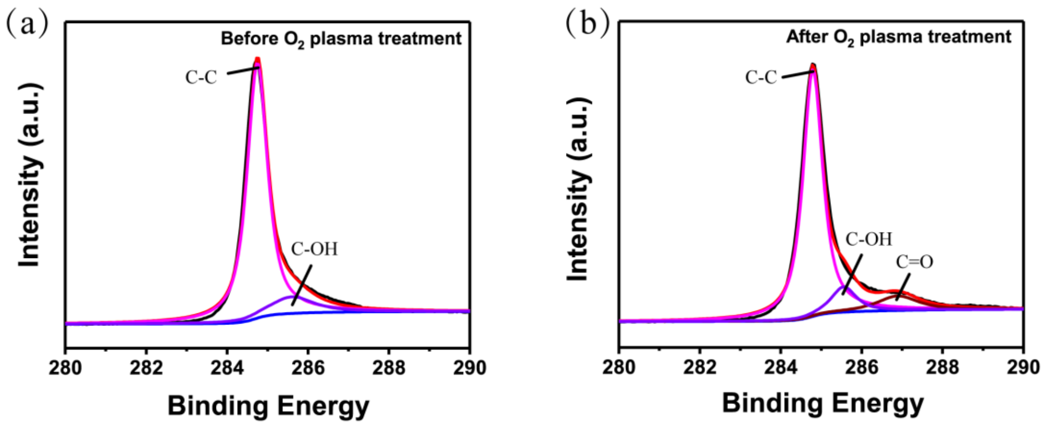

Figure S6. XPS spectrum of Au/CFP in the C region (a) before and (b) after O_2 treatment.

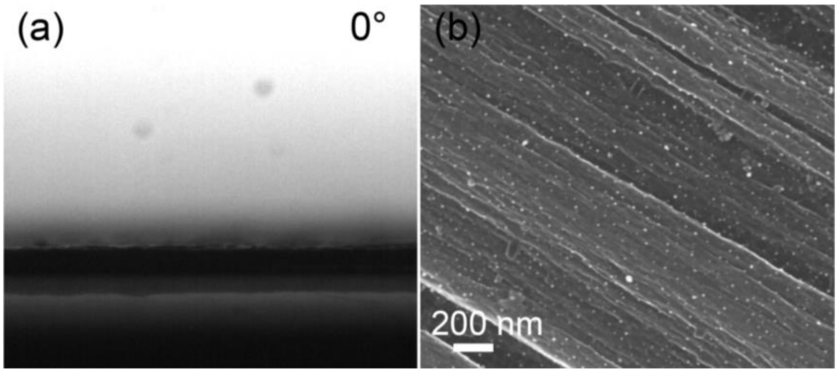

Figure S7. (a) The contact angle of Au/i-CFP and (b) The SEM image of Au/i-CFP.

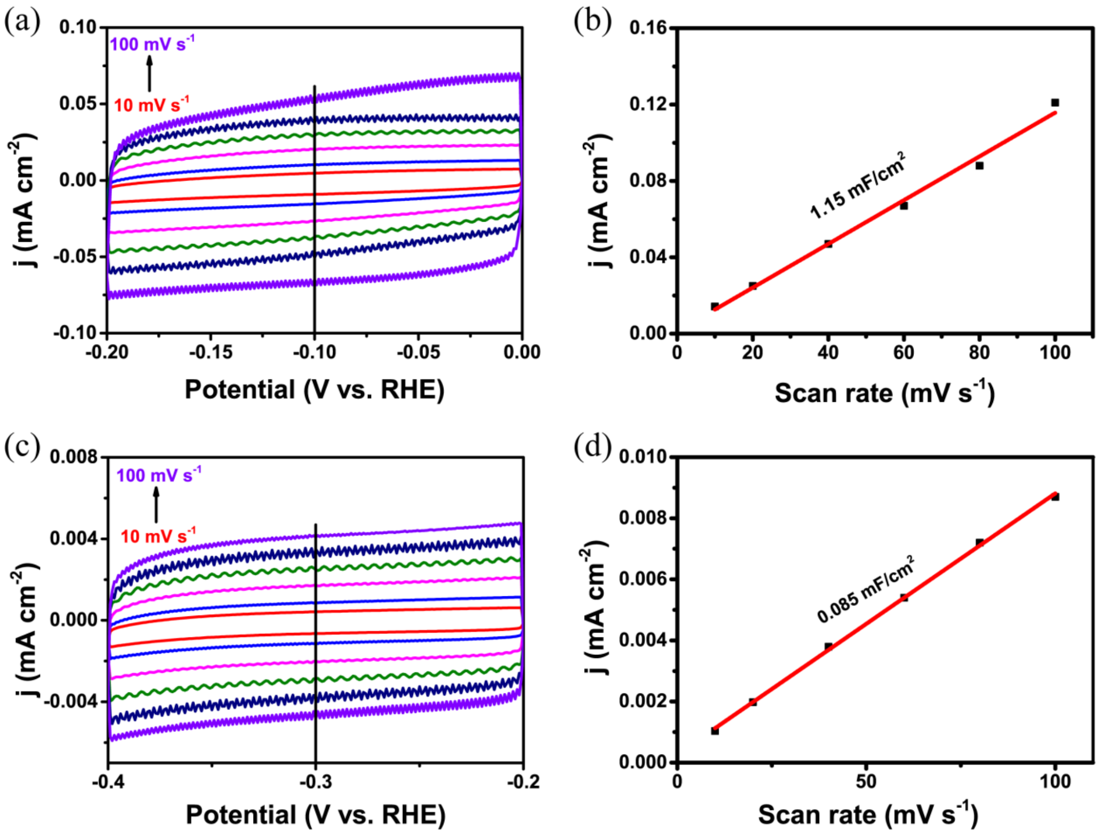

Figure S8. CV curves of Au/i-CFP (a) and Au/o-CFP (c) in the range of -0.2 and -0.4 V under Ar condition. Capacitive current densities at -0.3 V derived from CV curves against scan rates for Au/i-CFP (b) and Au/i-CFP (d).

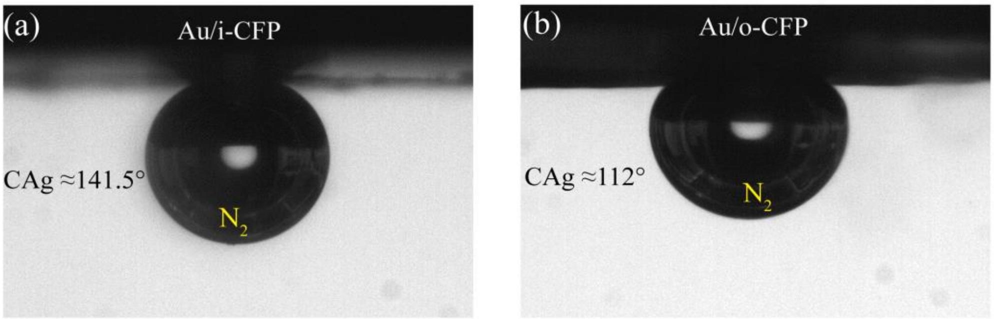

Figure S9. The shapes of N_2 bubble on the surface of Au/i-CFP (a) and Au /o-CFP (b) underwater.

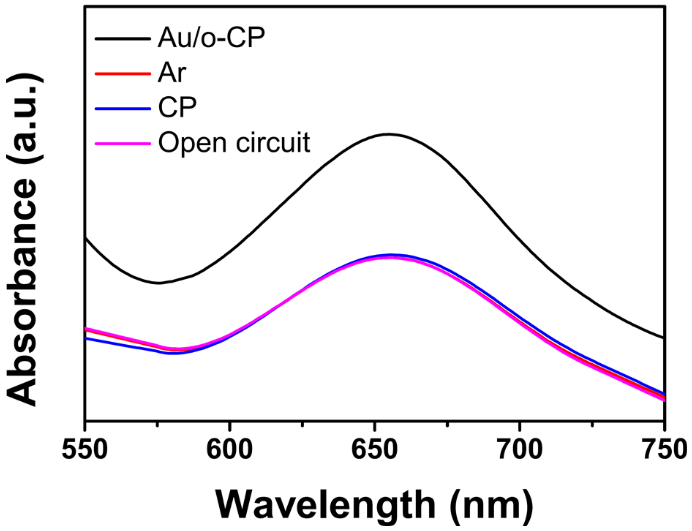

Figure S10. UV-Vis absorption spectra of the electrolytes stained with indophenols indicator under different conditions.

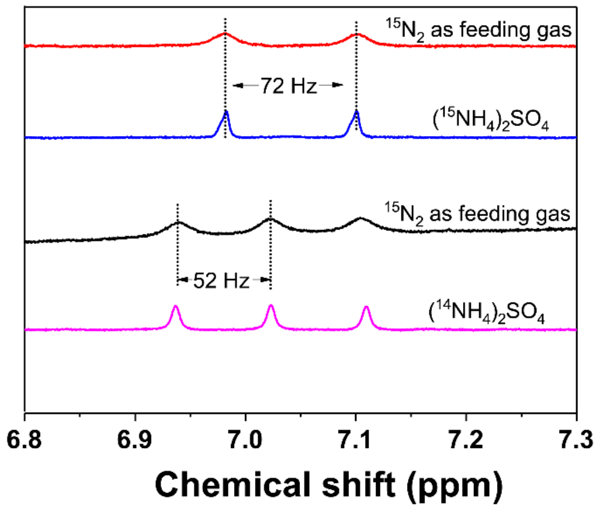

Figure S11. ^{15}N isotope labelled tests. ^{1}H NMR spectra for $(^{14}NH_4)_2SO_4$, $(^{14}NH_4)_2SO_4$ and the electrolyte after 6-h electrolysis with $^{14}N_2$ and $^{15}N_2$ as the feeding gases.

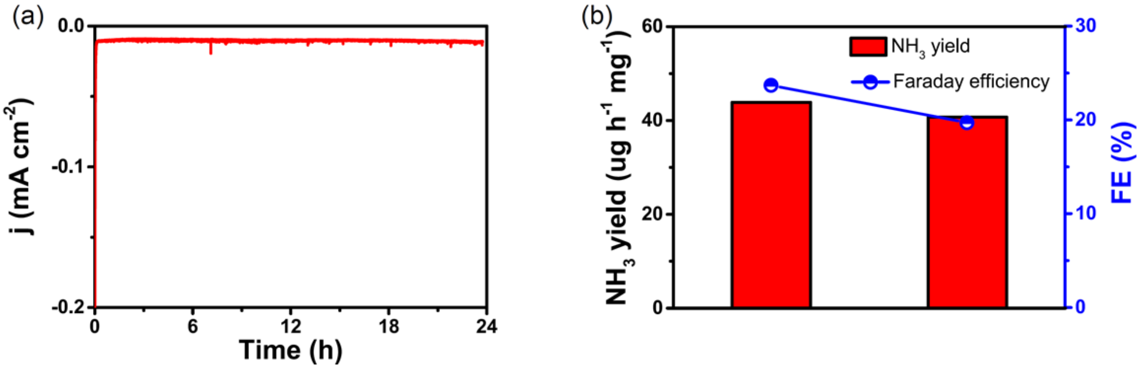

Figure S12. (a) The long time stability test of Au/o-CFP for 24 h and (b) corresponding their NH_3 yield rate and FE.

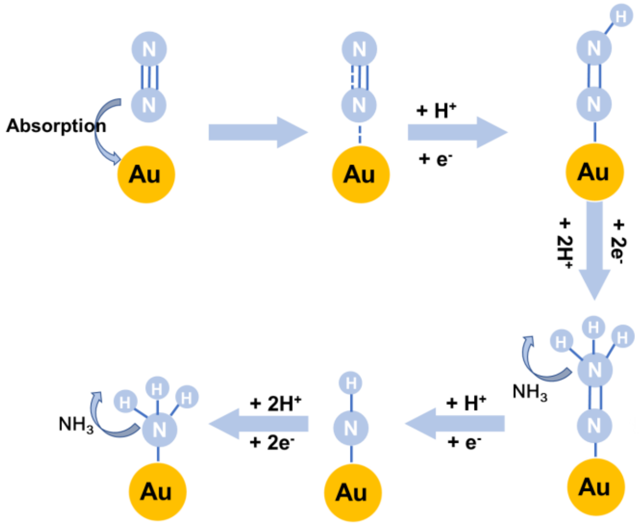

Figure S13. The possible NRR pathway for NRR on the Au surface.

Table S1. Comparison of the NRR performance for Au/o-CFP with recently reported NRR electrocatalysts under ambient conditions.

Catalyst	Electrolyte	NH_3 yield rate	FE (%)	Ref.
**Au/o-CFP**	**0.1 M Na_2SO_4**	**40.6 μg h^{-1} $mg^{-1}_{cat.}$**	**31.3**	**This work**
Cu/TiO_2	0.5 M $LiClO_4$	21.31 μg h^{-1} $mg^{-1}_{cat.}$	21.99	[1]
B-Ag NSs	0.1 M HCl	26.48 μg h^{-1} $mg^{-1}_{cat.}$	8.86	[2]
Au-TiO_2 sub-nanocluster	0.1 M HCl	21.4 μg h^{-1} $mg^{-1}_{cat.}$	8.11	[3]
Porous Au/Ni foam	0.1 M Na_2SO_4	29.43 μg h^{-1} $mg^{-1}_{cat.}$	13.36	[4]
Au/Ti_3C_2	0.1 M Na_2SO_4	23 μg h^{-1} $mg^{-1}_{cat.}$	34	[5]
Nanoporous Au@ZIF	0.1 M Na_2SO_4	28.7 μg h^{-1} $mg^{-1}_{cat.}$	44	[6]
PdRu tripods	0.1 M KOH	37.23 μg h^{-1} $mg^{-1}_{cat.}$	1.85	[7]
α-Au/CeOx-RGO	0.1 M HCl	8.3 μg h^{-1} $mg^{-1}_{cat.}$	10.10	[8]

Au flower	0.1 M HCl	25.57 $\mu g\ h^{-1}\ mg^{-1}_{cat.}$	6.05	[9]
PdRu BPNs	0.1 M HCl	25.92 $\mu g\ h^{-1}\ mg^{-1}_{cat.}$	1.53	[10]
Ru SAs/g-C_3N_4	0.5 M NaOH	23.0 $\mu g\ h^{-1}\ mg^{-1}_{cat.}$	8.3	[11]
Body－centered cubic PdCu	0.5 M LiCl	35.7 $\mu g\ h^{-1}\ mg^{-1}_{cat.}$	11.5	[12]
PdO/Pd	0.1 M NaOH	18.2 $\mu g\ h^{-1}\ mg^{-1}_{cat.}$	11.5	[13]
Ag_3Cu	0.1 M Na_2SO_4	24.59 $\mu g\ h^{-1}\ mg^{-1}_{cat.}$	13.28	[14]

Table S2. The average loading mass of Au NPs with different size detected by ICP-MS.

Samples	Au (6.76 nm)	Au (14.2 nm)	Au (21.9 nm)
Average loading mass (μg)	12.767	30.760	55.06

References

[1] T. W. Wu, H. T. Zhao, X. J. Zhu, Z. Xing, Q. Liu, T. Liu, S. Y. Gao, S. Y. Lu, G. Chen, Abdullah M. Asiri, Y. N. Zhang, X. P. Sun, *Adv. Mater.* **2020**, 2000299.

[2] Y. Li, H. Yu, Z. Wang, S. Liu, Y. Xu, X. Li, L. Wang, H. Wang, *Chem. Commun.* **2019**, 55, 14745.

[3] M. M. Shi, D. Bao, B. R. Wulan, Y. H. Li, Y. F. Zhang, J. M. Yan, Q. Jiang, *Adv. Mater.* **2017**, 29, 1606550.

[4] H. Wang, H. Yu, Z. Wang, Y. Li, Y. Xu, X. Li, H. Xue, L. Wang, *Small* **2019**, 15, 1804769.

[5] D. Liu, G. Zhang, Q. Ji, Y. Zhang, J. Li, *ACS Appl. Mater. Interfaces* **2019**, 11, 25758.

[6] Y. Yang, S. Q. Wang, H. Wen, T. Ye, J. Chen, C. P. Li, M. Du, *Angew. Chem. Int. Ed.* **2019**, 131, 15506.

[7] H. Wang, Y. Li, C. Li, K. Deng, Z. Wang, Y. Xu, X. Li, H. Xue, L. Wang, *J. Mater. Chem. A* **2019**, 7, 801.

[8] S. J. Li, D. Bao, M. M. Shi, B. R. Wulan, J. M. Yan, Q. Jiang, *Adv. Mater***.** **2017**, 29, 1700001.

[9] Z. Wang, Y. Li, H. Yu, Y. Xu, H. Xue, X. Li, H. Wang, L. Wang, *ChemSus*C*hem* **2018**, 11, 3480.

[10] Z. Wang, C. Li, K. Deng, Y. Xu, H. Xue, X. Li, L. Wang, H. Wang, *ACS Sustain. Chem. Eng.* **2018**, 7, 2400.

[11] B. Yu, H. Li, J. White, S. Donne, J. Yi, S. Xi, Y. Fu, G. Henkelman, H. Yu, Z. Chen, *Adv. Funct. Mater.* **2019**, 1905665.

[12] W. Tong, B. Huang, P. Wang, L. Li, Q. Shao, X. Huang, *Angew. Chem. Int. Ed.* **2019**, DOI:10.1002/anie.201913122.

[13] J. Lv, S. Wu, Z. Tian, Y. Ye, J. Liu, C. Liang, *J. Mater. Chem. A* **2019**, 7, 12627.

[14] H. Yu, Z. Wang, D. Yang, X. Qian, Y. Xu, X. Li, H. Wang, L. Wang, *J. Mater. Chem. A* **2019**, 7, 12526.
